# Iron Handling in Tumor-Associated Macrophages—Is There a New Role for Lipocalin-2?

**DOI:** 10.3389/fimmu.2017.01171

**Published:** 2017-09-20

**Authors:** Michaela Jung, Andreas Weigert, Christina Mertens, Claudia Rehwald, Bernhard Brüne

**Affiliations:** ^1^Faculty of Medicine, Institute of Biochemistry I, Goethe University Frankfurt, Frankfurt, Germany; ^2^Faculty 15, Biological Sciences, Goethe University Frankfurt, Frankfurt, Germany; ^3^Project Group Translational Medicine and Pharmacology TMP, Fraunhofer Institute for Molecular Biology and Applied Ecology, IME, Frankfurt, Germany

**Keywords:** apoptosis, phagocytosis, macrophage polarization, sphingosine-1-phosphate, lipocalin-2, tumor progression

## Abstract

Carcinogenesis is a multistep process. Besides somatic mutations in tumor cells, stroma-associated immunity is a major regulator of tumor growth. Tumor cells produce and secrete diverse mediators to create a local microenvironment that supports their own survival and growth. It is becoming apparent that iron acquisition, storage, and release in tumor cells is different from healthy counterparts. It is also appreciated that macrophages in the tumor microenvironment acquire a tumor-supportive, anti-inflammatory phenotype that promotes tumor cell proliferation, angiogenesis, and metastasis. Apparently, this behavior is attributed, at least in part, to the ability of macrophages to support tumor cells with iron. Polarization of macrophages by apoptotic tumor cells shifts the profile of genes involved in iron metabolism from an iron sequestering to an iron-release phenotype. Iron release from macrophages is supposed to be facilitated by ferroportin. However, lipid mediators such as sphingosine-1-phosphate, released form apoptotic tumor cells, upregulate lipocalin-2 (Lcn-2) in macrophages. This protein is known to bind siderophore-complexed iron and thus, may participate in iron transport in the tumor microenvironment. We describe how macrophages handle iron in the tumor microenvironment, discuss the relevance of an iron-release macrophage phenotype for tumor progression, and propose a new role for Lcn-2 in tumor-associated macrophages.

## Macrophage Uptake of Dying Cells in the Tumor Microenvironment

Among factors of the tumor microenvironment that shapes the macrophage phenotype to promote cancer are dying cells (1). Tumor cells and other tumor-resident cells undergoing programmed, apoptotic, or necroptotic, as well as accidental necrotic cell death are sensed and removed by macrophages, which induces different functional macrophage programs (Table 1). While lytic forms of cell death such as necroptosis and necrosis predominantly induce inflammatory cascades that may promote tumor initiation through modifying DNA and triggering cytokine-induced survival pathways in tumor cells (2), apoptotic cells (AC) induce macrophage-dependent matrix remodeling, recruitment of vasculature, and inhibition of antitumor inflammation (1, 3). These properties of AC are seen in analogy to their function during wound healing and regeneration (4, 5), supporting the notion that tumors are “wounds that do not heal” (6). The interaction of macrophages and dying cells, however, does not only alter their functional response, it also comes with a high metabolic load after engulfment of cell debris that needs to be handled by macrophages (7, 8). Hereby, macrophages can be considered an extravascular relay station of tumor-associated metabolism, to acquire and redistribute metabolic intermediates and other (bio)chemical substances, including iron, as outlined in more detail below (see Macrophage Subsets and Iron Handling). To our knowledge, there are no detailed studies comparing the metabolic challenges macrophages face when taking up apoptotic versus necroptotic or necrotic cells, and whether redistribution of nutrients such as iron differs in these circumstances. Studies toward these directions will help to aid decisions, which mode of cell death should be initiated in pathologies such as cancer.

**Table 1:** Molecules involved in the attraction, recognition, and polarization of phagocytes by dying cells.

Mode of cell death	Mode of interaction with phagocytes	Dying cell-derived molecules	Outcome/goal
Apoptosis	Attraction	ATP/UTP (9), LPC (10), S1P (11), RPS19 (12), EMAPII (13), CX3CL1 (14)	Early phagocyte recruitment
	Recognition	PS (15), CRT (16), ANXA1 (17), PTX3 (18)	Corpse removal (phagocytosis)
	Polarization	Tolerogenic apoptosis: PS (19), S1P (20), IL-38 (21), ANXA1 (17)	Immuno-suppression
	Polarization	Immunogenic apoptosis: CRT (16), ATP (22)	Immune activation

Necrosis	Attraction	Primary necrosis: ATP?	Phagocyte recruitment
		Secondary necrosis: ANXA1 (23)	
	Recognition	PS (24), complement (25), antibodies (25), pentraxins (25), F-actin (26)	Corpse removal (macropinocytosis)
	Polarization	HMGB1 (27), ATP (28), DNA (29), IL-1α (30), IL-33 (31)	Immune activation

Necroptosis	Attraction	ATP (32), others?	(Early) phagocyte recruitment
	Recognition	PS (33), others?	Corpse removal (mode unclear)
	Polarization	HMGB1, ATP, DNA, IL-1α, IL-33 + induced DAMPs? [reviewed in Ref. (34)]	Immune activation

*LPC, lysophosphatidylcholine; S1P, sphingosine-1-phosphate; RPS19, ribosomal protein S19; EMAPII, endothelial monocyte-activating polypeptide 2; PTX3, pentraxin 3; IL, interleukin; ANXA-1, annexin A1; CRT, calreticulin; PS, phosphatidylserine; HMGB1, high mobility group box 1; DAMP, damage-associated molecular pattern*.

## Macrophage Polarization by Dying Cells

Macrophage interactions with cells succumbed to different modes of cell death show overlapping and also discreet molecular features at the levels of attraction, recognition, and subsequent alteration of the macrophage phenotype (macrophage polarization) as summarized in Table 1.

In a first step, macrophages need to be alerted to their prey. In the case of AC, this is mediated by the active release of phagocyte-attracting molecules, so-called “find-me signals.” Their functions are intrinsically coupled to the apoptotic machinery, i.e., demanding caspase activation (35, 36). The release of find-me signals serves to recruit macrophages with the goal to efficiently clear apoptotic corpses before they undergo secondary necrosis. To achieve this goal, AC produce a variety of different find-me signals, probably dependent on the respective apoptotic stimulus. These include the lipids lysophosphatidylcholine and sphingosine-1-phosphate (S1P), the nucleotides ATP and UTP as well as the proteins fractalkine (CX3CL1), ribosomal protein S19, and endothelial monocyte-activating polypeptide 2. Moreover, apoptosis in the context of an inflammatory environment generates a number of different chemokines (3, 37–39). Of these, only CX3CL1 (14), ATP/UTP (9), and S1P (40) have been connected to phagocyte recruitment to AC *in vivo*. The diverse biochemical nature of these find-me signals and their different production kinetics (35) suggests a remarkable degree of redundancy. This redundancy likely ensures efficient macrophage recruitment at different time-points during the apoptotic cascade and from different locations, i.e., local macrophages versus monocytes from the circulation, based on short half-life versus long half-life of the find-me signals and their concentration in local tissues versus the circulation (14, 41, 42). When looking at lytic forms of cell death, characterized by the loss of plasma membrane integrity, the picture appears less clear. Secondary necrotic cells that were formerly apoptotic generate distinct find-me signals such as annexin A1 (ANXA1) fragments to sustain their clearance (23). Whether such ANXA1 fragments or other specific find-me signals are actively produced during necrosis or cells undergoing accidental, primary necrosis is largely unknown. A recent report suggests the release of nucleotides from necroptotic cells that, *in vitro*, induced a rapid and immunological silent clearance (32). Importantly, lytic cell death will promote the passive release of a number of apoptotic cell-derived find-me signals such as lipids or nucleotides, likely in higher quantities. The contribution of these molecules to necrotic/necroptotic cell clearance remains to be determined.

Regardless the find-me signal, phagocytes need to discriminate dying cells from their living neighbors. This is accomplished by the recognition of “eat-me” signals that are exposed by dying cells, in concert with the absence of “don’t eat-me” signals that restrict the uptake of living cells (43, 44). The most prominent eat-me signal that appears to be relevant to clear all dying cells, irrespective of the mode of cell death is the phospholipid phosphatidylserine (PS) that is confined to the inner leaflet of the plasma membrane in living cells, but gets oxidized and redistributed to the outer leaflet during apoptosis (15, 45, 46). PS is also involved in clearance of necrotic or necroptotic cells, but whether oxidative modification is equally required remains unknown (24, 25, 33). Other eat-me signals that are specifically exposed on the plasma membrane of AC include calreticulin (CRT), ANXA1, and the long pentraxin PTX3 (37). These eat-me signals are recognized either directly by specific receptors including scavenger receptors, complement receptors, C-type lectin receptors, a number of PS-specific receptors, and the pattern recognition receptor (PRR) CD14, or indirectly *via* bridging molecules that mainly promote the recognition of PS by Tyro, Axl, and MerTK-family receptor tyrosine kinases or the vitronectin receptor (VnR, α_v_β_3_ integrin) (3, 47). Some of the molecular interactions that mediate apoptotic cell recognition are employed for efficient uptake of necrotic debris, but specific pathways were also identified. Necrotic cell uptake is mediated by the classical complement pathway (C1q) and the mannose pathway (mannose-binding lectin and ficolins) recognized *via* complement receptors on phagocytes, antibodies, short pentraxins such as CRP, and serum amyloid protein *via* Fc-receptors, and also *via* PS recognition by VnR [reviewed in Ref. (25)]. Moreover, F-actin filaments in necrotic debris are recognized by the c-type lectin CLEC9A to facilitate clearance (26). The combination of different uptake receptors results in fundamental difference in the uptake mode. Whereas AC are engulfed *via* phagocytosis that is sensitive to PI3K inhibition, necrotic debris is taken up by PI3K-independent macropinocytosis (48). Specific uptake receptors and uptake mechanisms for necroptotic cells are so far not described.

Signals emanating from these numerous interactions between the phagocyte and its prey initiate not only corpse engulfment but also powerfully modulate inflammatory responses of the phagocytes [reviewed in Ref. (49)]. These responses are often fundamentally different when comparing apoptosis with cell death modes that are characterized by the loss of plasma membrane integrity and the subsequent spilling of intracellular components into the extracellular space. Many of such intracellularly confined molecules are considered as danger signals that are sensed by pathogen recognition receptors (PRRs) on phagocytes that also sense microbial components, and therefore trigger pro-inflammatory pathways. Such damage-associated molecular patterns (DAMPs) comprise high mobility group box 1, a protein usually found in complex with chromatin in the nucleus that activates toll-like receptors (TLR)-2, 4, 9 and the receptor for advanced glycation end products, the interleukins IL-1α and IL-33, DNA that is recognized by TLRs, and ATP that activates purine receptors including P2RX7. These pathways may be also exploited to trigger antitumor immunity (34, 50–53). Similar ligand receptor interactions may be assumed when considering the effect of necroptotic cells on the functional phenotype of their phagocytes based on the lytic nature of necroptotic cell death (53). An interesting difference is that necroptosis, as a form of regulated cell death, allows the transcriptional upregulation of additional DAMPs such as heat-shock proteins (34).

In contrast to necrosis or necroptosis, apoptotic cell death is usually considered as anti-inflammatory or immunologically silent. This is triggered at least partially through the recognition of PS, since mice lacking non-PS eat-me signal receptors such as CD14, CD36, and α_v_β_3_ integrin do not show major signs of auto-inflammation (54, 55). PS recognition on AC by macrophages suppressed the production of inflammatory cytokines, dependent on the autocrine production of transforming growth factor-β, platelet-activating factor, and prostaglandin E_2_ (PGE_2_) (19). Inhibition of inflammatory cytokine release from macrophage interacting with AC was further linked to inhibition of the classical NF-κB pathway (p65/p50 heterodimers) through transcriptional repression *via* the nuclear hormone receptor peroxisome proliferator-activated receptor γ (PPARγ) (56–59), or through PPARγ-dependent upregulation of phagocytic receptors such as MerTK (60). MerTK activation in turn interferes with NF-κB signaling (61). Besides inhibiting NF-κB, PS recognition on AC reduced the formation of nitric oxide (NO) and NADPH oxidase-dependent reactive oxygen species in macrophages (62–64). Therapeutically, targeting PS in tumors induced inflammatory macrophage activation to suppress tumor growth and progression in prostate tumors (65). MerTK-deficient mice with autochthonous mammary carcinoma were protected from metastasis, which was initiated by the interaction of macrophages with AC during mammary gland involution after pregnancy (66). Thus, PS recognition creates a feed-forward loop to guarantee efficient corpse removal and blocks a number of inflammatory pathways. This likely promotes efficient and immunologically silent corpse removal during homeostasis, but is exploited by tumors to promote carcinogenesis.

Also soluble factors produced by AC contribute to limiting inflammation, including the find-me signals CX_3_CL1 and S1P (3, 40, 67). Besides, AC release signals that exclusively limit destructive inflammation. The IL-1 family receptor antagonist IL-38 was proteolytically processed and released from AC and specifically inhibited the generation of Th17 cells, which are associated with chronic inflammation (21). In conclusion, apoptosis induces the production of membrane-bound and soluble cues that serve to ensure immunologically silent clearance by phagocytes.

The question remains why necrosis retains its inflammatory potential despite PS recognition being part of the program to remove necrotic debris. One explanation is that other signaling pathways are activated in necrotic versus AC due to alternative PS receptor usage and associated signaling pathways. For instance, apoptotic cell recognition activates the PI3K pathway, which is critical to limit inflammation (68), whereas removal of necrotic debris does not require PI3K (48). Alternatively, the context of PS recognition may matter, as phagocytes face necrotic cell-derived DAMPs before or simultaneously when engaging PS. Along this line, apoptotic cell recognition under conditions of danger, indicated by, e.g., ER stress or the presence of pathogens, promotes inflammation rather than restricting it. For instance, infected AC trigger autoimmune T cell (Th17) generation (69). Also, the potentially immunogenic molecule CRT on the surface of AC, which requires autophagy or ER stress linked to the execution of apoptosis, is recognized through the LDL-receptor-related protein on phagocytes (16, 70, 71). The find-me signal ATP, besides ensuring corpse clearance, can bind P2X purinoceptor 7 (P2RX7) to induce activation of the NOD-Like receptor family, pyrin domain containing 3 (NLRP3) inflammasome and subsequent IL-1β release. These examples illustrate that apoptosis does not always restrict inflammation, which may explain why necrosis can trigger inflammation despite PS recognition. In wounds, this ensures that inflammation proceeds as long as pathogens or other noxa are present. In tumors, the response to apoptosis likely depends on the microenvironment. Immunosuppressive apoptosis is exploited by the tumor to fuel its growth (1), whereas induction of immunogenic cell death may be exploited therapeutically to initiate protective immunity (72).

## Iron Handling Proteins in Macrophages

**Figure 1:**
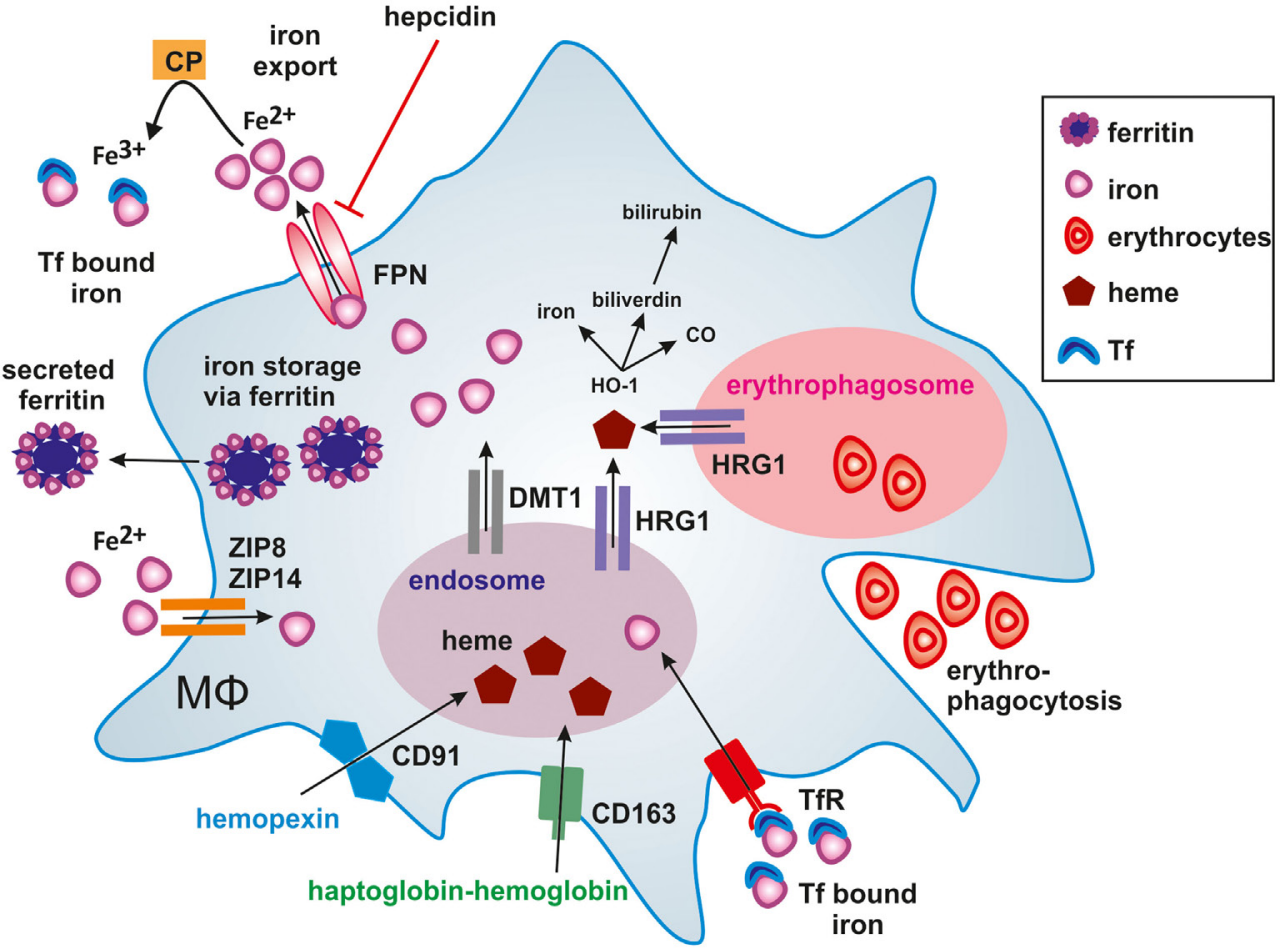
Iron handling in macrophages. Macrophages take up, metabolize, store, and export iron. Classically activated macrophages sequester iron by taking up transferrin (Tf) bound iron *via* the Tf-receptor (TfR) or accumulating ferrous iron (Fe^2+^) *via* zinc transporters ZIP8 and ZIP14. Iron export *via* ferroportin (FPN) is impaired by binding of hepcidin (HAMP), thus causing iron storage in ferritin. In alternatively activated macrophages, the uptake of hemopexin-heme (Hpx-heme) by CD91 or haptoglobin-hemoglobin by CD163 into endosomes as well as phagocytosis of senescent erythrocytes into erythrophagosomes results in the release of heme *via* the heme transporter HRG1 to the cytosol. The subsequent activation of heme oxygenase-1 (HO-1) further degrades heme into iron, CO, and biliverdin, which is further processed to bilirubin. Fe^2+^ is exported from alternatively activated macrophages through FPN and oxidized to ferric iron (Fe^3+^) iron by ceruloplasmin (CP), which is essential for efficient binding to Tf. Secretion of ferritin-bound iron represents an alternative route of iron export.

In humans, approximately 60% of total body iron is present in erythrocytes, bound to heme in hemoglobin (73). About 2 million senescent red blood cells (RBCs) are cleared per second from the circulation by tissue-specific macrophages (74). Senescent erythrocytes get recognized by macrophages due to alterations in the membrane protein Band 3 that only is displayed by aged erythrocytes (75, 76). Additionally, PS is exposed on the outer leaflet of the cell membrane (77) and membrane rigidity is increased to foster recognition by macrophages (78). A daily turnover of about 20 mg iron makes macrophages essential players in iron metabolism, as we only take up 1–2 mg iron with our daily nutrition. In order to fulfill their essential roles in systemic iron homeostasis, macrophages evolved a variety of pathways to take up, recycle, store, or release iron. The majority of iron is delivered by transferrin (Tf), the main iron-transport protein in the blood, circulating between the reticuloendothelial system, and the bone marrow in order to guarantee hematopoiesis. Tf binds to the transferrin receptor (TfR) and following internalization, iron is released from Tf in the endosome. Subsequently, the divalent metal transporter-1 (DMT-1) shuttles iron from the endosome into the cytoplasm. In addition, macrophages recycle phagolysosomal iron through natural resistance-associated macrophage protein 1 (Nramp-1), a divalent metal transporter homologous to DMT-1 (79). If iron supply exceeds its demands, iron can be stored by the iron storage protein ferritin, consisting of ferritin heavy chain (FTH) and ferritin light chain (FTL) subunits. FTH and FTL differ in their function, as FTH has a ferroxidase activity and FTL is important for iron core nucleation (80). By transforming soluble and reactive ferrous iron (Fe^2+^) of the cytoplasmic labile iron pool (LIP) into the insoluble ferric iron (Fe^3+^) and store Fe^3+^ within the soluble ferritin shell, cells avoid the potential damage of redox active iron, i.e., Fenton chemistry. Consequently, the intracellular iron amount must be tightly regulated. Iron is sensed by the iron-regulatory proteins (IRPs) IRP1 and IRP2. When intracellular iron is low, IRPs bind to iron responsive elements (IREs) in the untranslated regions (UTRs) of certain mRNAs. Binding of IRPs to IREs in the 5′-UTR attenuates translation, whereas binding to IREs in the 3′-UTR stabilizes respective mRNAs and fosters translation. Mechanistically, when iron is high, mRNAs of TfR and DMT-1 are unstable, which decreases iron uptake and transport. Simultaneously, iron storage is supported by releasing a translational blockade of FTH and FTL. Iron export from cells is achieved through ferroportin (FPN), the only known ferrous iron exporter. Although details of the transport remain unclear, the transport of ferrous iron (Fe^2+^) requires ferroxidase activity to convert it to ferric iron (Fe^+3^) in order to load it onto Tf. The oxidation to ferric iron is catalyzed by either free or GPI-anchored ceruloplasmin (CP) or the transmembrane protein hephestin that are copper-containing ferroxidases in order to stabilize FPN to facilitate iron efflux (81) and assure efficient Tf-loading. This makes FPN an important checkpoint to adjust global and local iron homeostasis and to adjust iron storage versus its release (82). The protein amount of FPN is controlled by the peptide hormone hepcidin, which primarily is produced in the liver. Hepcidin regulates FPN stability by inducing its internalization and proteasomal degradation thus, affecting the systemic iron level (83) by reducing iron export. Consequently, hepcidin expression and release from the liver increases with the systemic iron amount and decreases under iron deprived conditions to guarantee macrophage iron supply for erythropoiesis. This is crucial as iron supply is the rate-limiting step during erythropoiesis (84). As shown in Figure 1, heme bound to the heme-sequestering protein hemopexin (Hpx) is taken up by the hemopexin receptor (CD91), while CD163 binds haptoglobin-transported hemoglobin (85).

Overall, iron metabolism is tightly regulated by a network of proteins to guarantee iron homeostasis with specific macrophage subsets in key positions to fulfill their role in iron recycling by erythrophagocytosis and iron release to sustain erythropoiesis.

## Macrophage Subsets and Iron Handling

The major macrophages subsets involved in systemic iron homeostasis consist of red pulp macrophages in the spleen and Kupffer cells in the liver. Their main function is to phagocytose damaged or senescent erythrocytes to recover iron. Therefore, phagocytosed erythrocytes are exposed to reactive oxygen species and hydrolytic enzymes in the erythrolysosomal compartment, with the subsequent release of hemoglobin and heme. Heme is then degraded by heme oxygenase-1 (HO-1) to carbon monoxide, biliverdin, and free iron, which usually joins the chelatable LIP or is stored (86). Heme also induces the expression of the transcription factor Spi-C, which is essential for the differentiation of red pulp macrophages (87) and erythroid island macrophages in the bone marrow (88), with no other macrophage subset being affected. Consequently, Spi-C knockout mice accumulate iron in the red pulp of the spleen, as RBCs are trapped but inefficiently phagocytosed (87). Red pulp macrophages, compared to other macrophage subsets, show a specialized gene signature required for enhanced iron recycling in order to fulfill their crucial metabolic function in systemic iron homeostasis.

However, macrophages are highly plastic cells regarding their functional properties, responding to a great number of inflammatory stimuli (89, 90). As extremes within a continuum, two opposing states of macrophage activation were identified. Macrophages activated by T helper 1 (Th1) cell-derived interferon-γ (IFN-γ), in combination with TLR ligands such as lipopolysaccharide (LPS) creates cells with a strong pro-inflammatory profile. These “classically activated” macrophages generate pro-inflammatory mediators such tumor necrosis factor-α, IL-1β, IL-6, IL-12, and IL-23, reactive oxygen and nitrogen species, and present antigens to T cells. Classically activated macrophages are efficient in microbial host defense and show antitumor activity. In contrast, macrophages stimulated by activated T helper 2 (Th2) cell-derived IL-4 or IL-13, or by IL-10, produce alternative sets of cytokines, functionally oppose the repertoire of classically activated macrophages, and help to resolve inflammation. Additionally, such “alternatively activated” macrophages express specific phagocytic receptors, combat extracellular parasites, and help to promote tissue remodeling by producing extracellular matrix and growth factors (89–91). Taking their functional diversity into account, it is not surprising that macrophages also show distinct properties in handling iron (92). Iron recycling by macrophages comprises the steps of uptake, storage, and release. These are critical features, as there is no way to get rid of body iron, except during bleeding or sloughing of mucosa and/or skin. As part of their functional repertoire upon activation of tissue-resident macrophages or differentiation of newly recruited, tissue-infiltrating monocytes, macrophages evolved multiple ways to handle iron (Table 2) according to diverse microenvironmental stimuli.

**Table 2:** Iron regulated genes in classically and alternatively activated macrophages.

	Classically activated	Alternatively activated
**Receptors**		
Transferrin receptor (TfR)	↓	↑
CD91	↓	↑
CD163	↓	↑
**Recycling**		
Heme oxygenase-1 (HO-1)	↓	↑
**Trafficking**		
Ferroportin (FPN)	↓	↑
Divalent metal transporter 1 (DTM-1)	↑	↑
Transferrin	↑	↑
**Storage**		
Ferritin (FT)	↑	↓
**Regulation**		
Hepcidin (HAMP)	↑	↓
Iron-regulatory proteins (IRP)	↓	↑
**Oxidreductase**		
Ceruloplasmin (CP)	↓	↑

*Regulation of genes related to iron metabolism in classically versus alternatively activated macrophages (93, 94). ↑ upregulation, ↓ downregulation*.

The groups of Recalcati and Cairo (93) discovered that classically activated macrophages tend to accumulate iron, whereas alternatively activated macrophages provide recycled iron to their microenvironment. To do so, classically activated macrophages maximize iron uptake directly *via* the TfR, and indirectly *via* the Nramp-1 and DMT-1 as well as through storage by ferritin, whereas they downregulate FPN-mediated iron export. During inflammation, this serves to deplete invading pathogens from iron (95–99). In addition, bacteria-derived LPS and pro-inflammatory cytokines cause macrophages to express hepcidin (83), which degrades FPN and adds to restrain iron. Thus, under infectious/inflammatory conditions, macrophages are a major site for storing iron. Acute phase proteins as well as the formation of reactive oxygen species and NO join to induce a macrophage iron-sequestration phenotype, mainly achieved by downregulating FPN. Opposed to these functions, alternatively activated macrophages provide iron to their local microenvironment (93, 94). Taking into account that alternatively activated macrophages express scavenger receptors that not only serve as PRRs but also sense and clear AC these macrophages accumulate hemoglobin during hemodialysis or inflammation. The inflammatory-promoting actions of free heme are antagonized (100–102), thereby fostering the resolution of inflammation. Moreover, the redox-sensitive transcription factor nuclear factor erythoid 2-like 2 gets activated with concomitant transcription of the iron exporter FPN and the heme-degrading enzyme HO-1 (40). In alternatively activated macrophages, heme-recycled iron joins the LIP for a rapid release *via* FPN, while in inflammatory macrophages iron is stored in ferritin. Iron export may also add to stabilize hypoxia-inducible factor (HIF) in macrophages, as iron is a prerequisite for the activity prolyl hydroxylases (PHD) as part of the HIF-degrading machinery (103). Lowering iron deactivates PHD enzymes, which stabilizes HIF-1α (104). Active HIF-1 causes target gene activation of, e.g., arginase-1, which are part of the alternative macrophage signature (105). These considerations suggest that in alternatively activated macrophages iron from the LIP is preferentially provided to the local microenvironment. In turn, this source of extracellular iron, provided by macrophages, may add to promote tissue regeneration, but may also be part of the tumor-promoting capacity of tumor-associated macrophages (TAMs). Mechanistically, an increased pool of iron stimulates proliferation of fibroblasts or tumor cells in the neighborhood of macrophages. The question remains how cancer cells acquire iron from their local microenvironment that includes, among others, macrophages. Conclusively, macrophages residing in the tumor stroma may provide iron to their local microenvironment, which includes the expression of alternative iron-transport mechanisms.

## TAMs and Iron in the Tumor Microenvironment

**Figure 2:**
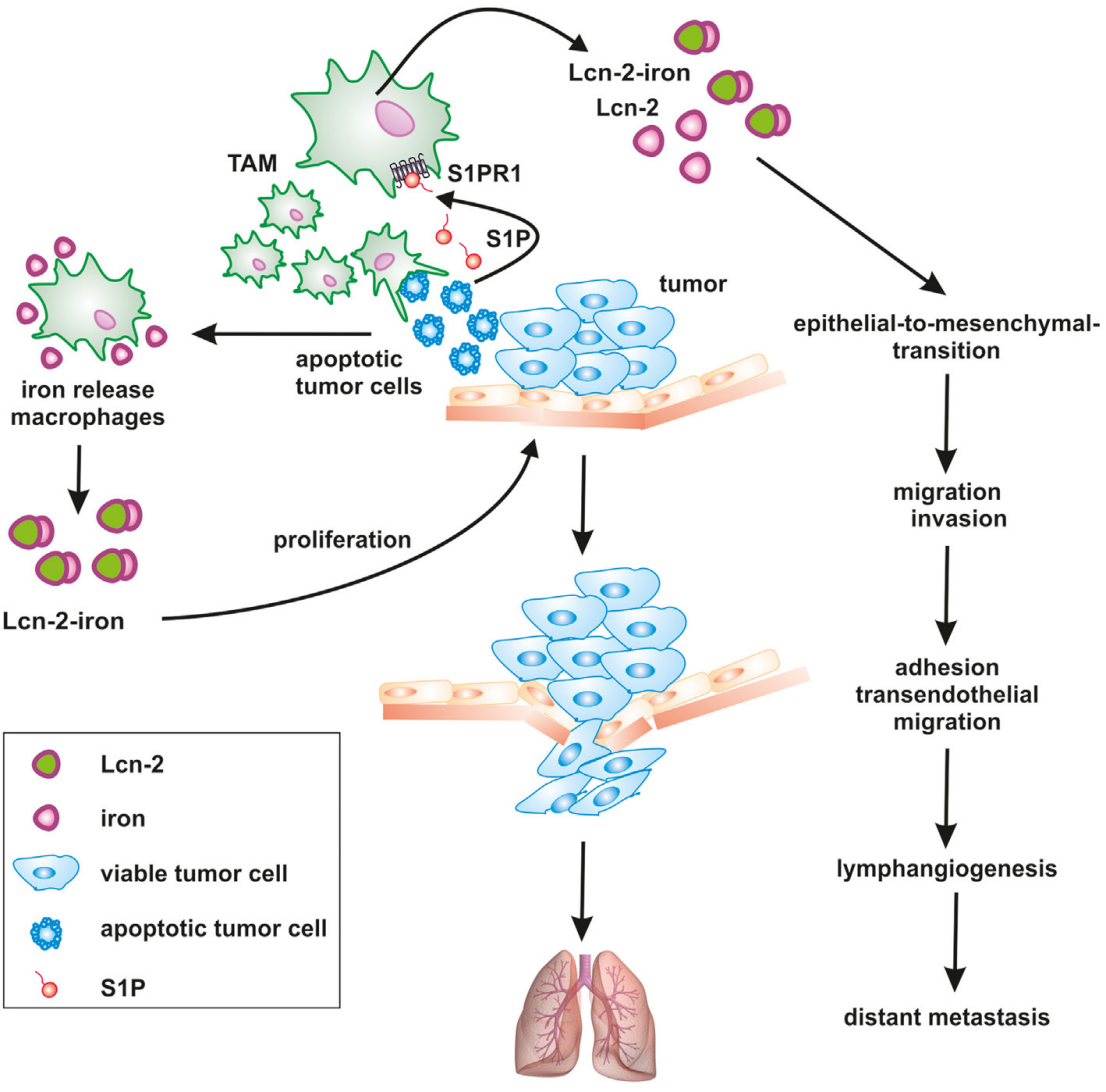
Apoptotic cell-derived S1P induces lipocalin-2 (Lcn-2) to promote tumor progression. The cross talk between apoptotic tumor cells and macrophages creates a feed-forward mechanism, where macrophages become polarized toward a pro-tumorigenic, iron-release phenotype, including the formation of iron-loaded Lcn-2. Lcn-2 is activated in tumor-associated macrophages (TAMs) through apoptotic cell-derived sphingosine-1-phosphate (S1P) downstream of the S1P receptor 1. TAM-derived, iron-loaded Lcn-2 exerts pro-tumorigenic actions by promoting tumor cell proliferation and enhancing metastatic spread to the lung at various steps, including the induction of epithelial-to-mesenchymal transition, migration, and invasion as well as adhesion and transendothelial migration.

Cell division, growth, and survival of malignant cells require iron. Therefore, tumor cells enhance iron import and storage mechanisms and decrease iron export. It is already known that tumor cells adopt an iron-utilization phenotype, tightly linked to intracellular iron sequestration (106). This is achieved by upregulating the TfR (107) and hepcidin (108) as well as downregulating FPN (109). In contrast to tumor cells, inflammatory cells of the stroma, e.g., infiltrating macrophages and lymphocytes, acquire an “iron-donor” phenotype (110), which is accomplished by upregulating the iron exporter FPN. Mechanistically, our and other labs noticed that IL-10- or IL-4-stimulated macrophages export iron to accelerate tumor growth by supplying iron to actively proliferating tumor cells (93, 94). This may point to a so far unappreciated facet of stromal cells in promoting tumor progression by supplying iron in order to facilitate the transition from pre-malignant lesions to invasive tumors. It was also speculated that tumor cells specifically hijack the process of erythrophagocytosis by macrophages to sustain their survival and proliferation. Knutson and coworkers illustrated the role of FPN in J774 macrophages in releasing ^59^Fe after phagocytosis of ^59^Fe-labeled RBCs (111). Erythrophagocytosis not necessarily is restricted to the systemic level, but may also occur locally under conditions of increased blood flow. In this respect, tumor cells may benefit from tumor angiogenesis and an increased migration of cells into tumor tissue (112), which fosters the convergence of TAMs with erythrocytes. Of note, tumor vessels are often leaky and it can be speculated that macrophages residing close to these vessels are particularly prone to recycle and donate iron to tumor cells (113). So far it remains unclear whether the physiological process of erythrophagocytosis alters genes that regulate iron metabolism in a way similar to those seen in alternatively activated macrophages or TAMs. TAMs alter their gene expression profile in favor of a tumor-supportive, iron-release phenotype (Figure 2). This is reflected by an increased expression of CD163, the high-affinity scavenger receptor for haptoglobin bound to hemoglobin (114). Subsequent to the uptake of hemoglobin or haptoglobin, released heme is then degraded *via* HO-1 and induces the downregulation of the transcription factor Bach-1, thereby allowing the transcription of FPN (115). Thus, at least in macrophages, heme may work as a modulator of FPN expression, independent of hepcidin. As a substantial part of plasma iron is provided by macrophages, recycled from senescent RBCs, it is surprising that mice carrying a knockout of FPN in macrophages only showed mild signs of anemia (116). One explanation could be that macrophages can export heme *via* the feline leukemia virus C receptor transporter (117), which might substitute for a loss of FPN. Moreover, in an inflammatory environment, ferritin can be secreted from lymphocytes and macrophages in the tumor stroma (118, 119). In line, extracellular ferritin causes proliferation of breast cancer cells, independent of its iron content. Another explanation, as discussed below, might be the development of additional or alternative iron-transport mechanisms.

## Lcn-2 may Function as an Iron Transporter in the Tumor Microenvironment

Taking into consideration that cancer cells have a higher demand for iron, it is tempting to speculate that tumor cells hijack macrophages to turn them into an iron-delivery cell. In this regard, we noticed that the high-affinity iron-carrier Lcn-2 is part of the pro-tumor macrophage phenotype and upregulated following macrophage-apoptotic cell interactions.

Lipocalin-2 belongs to the lipocalin superfamily. These proteins are known for their bacteriostatic effects by capturing and depleting siderophores (120). Furthermore, recent evidence suggests that Lcn-2 stimulates growth and differentiation in various cells (121). Exogenous Lcn-2 causes marker gene expression profiles that reflect early epithelial progenitors and epithelial cell proliferation (122). Our work revealed that apoptotic tumor cells stimulated protein expression and secretion of Lcn-2 in macrophages along with their functional shift toward an alternative phenotype (123, 124). Macrophage-derived Lcn-2 stimulates cancer cell proliferation (124), tumor cell dissemination, metastasis (125), and tumor lymphangiogenesis (126). Mice lacking Lcn-2 developed significantly less tumors, while an impact on metastases was not consistently observed (127–129). The impact of Lcn-2 on metastasis might depend on the cellular source, with at least macrophage-derived Lcn2 promoting metastasis in mammary carcinoma (125, 126). Studies in humans pointed to Lcn-2 as a pro-tumorigenic factor in breast cancer, correlated with decreased survival and reduced responsiveness to neoadjuvant chemotherapy (130, 131). Mechanistically, overexpression of Lcn-2 in non-invasive human MCF-7 breast cancer cells elicits an aggressive phenotype that promotes growth and metastasis by inducing epithelial-to-mesenchymal transition (132). So far most studies focused on the role of Lcn-2 in stabilizing matrix metalloproteinase-9 to explain cancer metastasis, linked to extracellular matrix degradation, migration, and invasion. Furthermore, Lcn-2 coordinates the expression of vascular endothelial growth factor and causes the induction of angiogenesis in the tumor microenvironment (133). Interestingly, Lcn-2 was mainly examined in tumor cells. The possibility that Lcn-2 is also provided by tumor-infiltrating immune cells was not fully appreciated. However, own results provided evidence that TAM express increasing amounts of Lcn-2 (124, 134).

We previously showed that the interaction of macrophages with AC shapes the macrophage phenotype and function (11). Importantly, macrophage activation upon their interaction with AC was independent of phagocytosis or cell-cell contact, but demanded the release of S1P from AC (135). Moreover, we demonstrated a critical involvement of sphingosine kinase 2 in the production of S1P during tumor cell apoptosis (20). We further obtained evidence that Lcn-2 was expressed in primary human macrophages in response to dying MCF-7 breast cancer cells (123). Mechanistically, Lcn-2 production was connected to S1P release from apoptotic cancer cells. S1P elicited signal transducer and activator of transcription 3-dependent induction of Lcn-2 in macrophages. siRNA studies in primary human macrophages and the use of bone marrow-derived macrophages from S1P receptor knockout mice suggested that the S1PR1 was required for Lcn-2 induction in macrophages (126). We substantiated Lcn-2 as a key macrophage phenotype determinant, with parallel actions during physiological tissue regeneration and repair mechanisms (123), but also under pathophysiological conditions such as tumor development. In human and experimental tumors, tumor-infiltrating macrophages are massively exposed to apoptotic tumor cells, since cells at core tumor regions undergo cell death as a consequence of oxygen and nutrient deprivation. Therefore, we speculate that dying tumor cells educate macrophages at core tumor regions in order to access additional iron *via* Lcn-2. However, it is presently unclear whether the pro-tumor actions of Lcn-2 depend on its iron loading or not. Previously, it was shown that iron-loaded holo-Lcn-2 favors cellular survival and proliferation by increasing the intracellular iron content and the induction of Bcl-2 (136). In contrast, the uptake to iron-free apo-Lcn-2 causes cell death, which was correlated to the expression of Bim.

Importantly, Lcn-2 does not directly bind iron. The iron-trafficking function of Lcn-2 largely depends on its association with bacterial or mammalian siderophores. Siderophores are iron-chelating molecules that were first described in bacteria (137–139). Devireddy et al. recently reported that mammals also produce iron-sequestring agents to enhance innate immune responses. Along this line, the mammalian siderophore 2,5-dihydroxybenzoic acid (2,5-DHBA) was characterized (140), which is structurally similar to the bacterial enterobactin. Lcn-2 interacts with siderophores in order to control bacterial growth as part of the innate immune response. Consequently, mice lacking Lcn-2 are more prone to a number of pathogens (141). Since siderophore-binding constitutes the limiting factor for Lcn-2-dependent iron handling, it is important to understand the function, regulation, and sources of mammalian siderophores. Several of the biological functions of Lcn-2 have already been linked to its association with the iron-loaded siderophore 2,5-DHBA (136). In the tumor, it might be speculated that tumor cells evolved a strategy to produce and secrete siderophores in order to sequester iron. Consequently, siderophore shuttling from tumor cells to TAM would allow Lcn-2 iron loading and the reverse transport of iron-loaded Lcn-2. Up to now, it is unclear how siderophores are taken up by mammalian cells. It also remains unknown whether 2,5-DHBA acts alone as the iron-chelating siderophore or whether it functions as the iron-binding moiety of a more complex siderophore structure as described for 2,3-DHBA in enterobactin. Regarding its production in mammalian cells, it was previously described that the mammalian enzyme 3-hydroxybutyrate dehydrogenase, type 2 (BDH2) synthesizes 2,5-DHBA. Unfortunately, the exact mechanism how BDH2 synthesizes 2,5-DHBA still remains elusive but the knockdown of BDH2 completely depleted cellular 2,5-DHBA (140). BDH2 knockout mice developed severe anemia and splenic iron overload (142) thus, confirming the requirement of 2,5-DHBA for iron transport (140). Additionally, the knockdown of BDH2 in mammalian cells points to an important role of 2,5-DHBA in balancing the LIP. A knockdown of BDH2 is linked to high cytoplasmic iron content and elevated levels of reactive oxygen species, whereas mitochondria became iron deficient (140). This increases an oxidative stress signature (143). Intriguingly, the expression of BDH2 negatively correlated with patient survival suffering from normal acute myeloid leukemia (144). However, regarding the clinical importance of siderophores, especially in tumors, more investigations are needed. Independent of their putative endogenous roles in pathology, sidrophores represent an attractive target for therapeutic approaches, e.g., as iron-chelating drugs in cancer therapy or iron-overload diseases, due to their high iron affinity (145, 146). Another possibility would be the use of siderophores as “trojan horse” in order to deliver antibiotics or other toxic compounds to resistant bacteria (147) and possibly tumor cells.

## Conclusion

In the tumor microenvironment, macrophages are subjected to an intense cross talk with tumor cells. Signal exchange is facilitated by chemically diverse, soluble mediators as well as communication by cell-cell contacts. This comprises the release of S1P from apoptotic tumor cells. As a consequence of the liaison between innate immune and tumor cells, the phenotype of macrophages changes. They become less cytotoxic and their cytokine mediator profile supports rather than antagonizes tumor progression to basically support all hallmarks of cancer (Figure 2).

Tumor cells with their high capacity to proliferate show a strong demand for accumulating iron. Consequently, it seems rational that TAMs gain an iron-release phenotype, thereby allowing tumor cells to access additional sources of iron. In the tumor context, macrophages may be forced by S1P to upregulate a so far unappreciated iron export system, the key component being Lcn-2. Although highly speculative, one can envision that the siderophore 2,5-DHBA is produced and released from tumor cells, travels to macrophages to load iron and in turn shuttles back to tumor cells to unload its cargo. Surplus iron in tumor cells is now being used to foster growth and survival and to add to the distinct phases of tumor dissemination and metastasis. This unique iron distribution system may offer the advantage to interfere pharmacologically and thus, more selective than manipulating the overall iron homeostasis in our body. Selectivity may be obtained if we successfully chelate iron in TAMs, interfere with expression regulation of macrophage Lcn-2 or the proposed shuttling of the mammalian siderophore.

While the overarching role of TAMs during tumor progression is undisputed, underlying molecular mechanisms are less clear. We believe that altering mechanisms of iron handling in tumor and stroma cells, i.e., macrophages has to be added to the list of changes that occur in the tumor microenvironment and shape the unique macrophage phenotype found in tumors.

## Author Contributions

All authors added to designing the work, acquired, analyzed, and interpreted data, wrote parts of the manuscript, approved the final version, and discussed all questions concerning the accuracy and integrity of the work.

## Conflict of Interest Statement

The authors declare that the research was conducted in the absence of any commercial or financial relationships that could be construed as a potential conflict of interest.

## Abbreviations

ANXA1, annexin A1; BDH2, 3-hydroxybutyrate dehydrogenase, type 2; CX3CL1, fractalkine; DAMP, damage-associated molecular pattern; DHBA, dihydroxybenzoic acid; DMT-1, divalent metal transporter-1; FTH, ferritin heavy chain; FTL, ferritin light chain; FPN, ferroportin; HIF, hypoxia-inducible factor; HO-1, heme oxygenase-1; IL, interleukin; IRE, iron responsive element; IRP, iron-regulatory protein; Lcn-2, lipocalin-2; PS, phosphatidylserine; TAM, tumor-associated macrophage; Tf, transferring; TfR, transferrin receptor; S1P, sphingosine-1-phosphate.
